# Dissecting Molecular Differences between Wnt Coreceptors LRP5 and LRP6

**DOI:** 10.1371/journal.pone.0023537

**Published:** 2011-08-24

**Authors:** Bryan T. MacDonald, Mikhail V. Semenov, He Huang, Xi He

**Affiliations:** F. M. Kirby Neurobiology Center, Children's Hospital, Harvard Medical School, Boston, Massachusetts, United States of America; University of Washington, United States of America

## Abstract

Low-density lipoprotein receptor-related proteins 5 and 6 (LRP5 and LRP6) serve as Wnt co-receptors for the canonical β-catenin pathway. While LRP6 is essential for embryogenesis, both LRP5 and LRP6 play critical roles for skeletal remodeling, osteoporosis pathogenesis and cancer formation, making LRP5 and LRP6 key therapeutic targets for cancer and disease treatment. LRP5 and LRP6 each contain in the cytoplasmic domain five conserved PPPSPxS motifs that are pivotal for signaling and serve collectively as phosphorylation-dependent docking sites for the scaffolding protein Axin. However existing data suggest that LRP6 is more effective than LRP5 in transducing the Wnt signal. To understand the molecular basis that accounts for the different signaling activity of LRP5 and LRP6, we generated a series of chimeric receptors via swapping LRP5 and LRP6 cytoplasmic domains, LRP5C and LRP6C, and studied their Wnt signaling activity using biochemical and functional assays. We demonstrate that LRP6C exhibits strong signaling activity while LRP5C is much less active in cells. Recombinant LRP5C and LRP6C upon in vitro phosphorylation exhibit similar Axin-binding capability, suggesting that LRP5 and LRP6 differ in vivo at a step prior to Axin-binding, likely at receiving phosphorylation. We identified between the two most carboxyl PPPSPxS motifs an intervening “gap4” region that appears to account for much of the difference between LRP5C and LRP6C, and showed that alterations in this region are sufficient to enhance LRP5 PPPSPxS phosphorylation and signaling to levels comparable to LRP6 in cells. In addition we provide evidence that binding of phosphorylated LRP5 or LRP6 to Axin is likely direct and does not require the GSK3 kinase as a bridging intermediate as has been proposed. Our studies therefore uncover a new and important molecular tuning mechanism for differential regulation of LRP5 and LRP6 phosphorylation and signaling activity.

## Introduction

The Wnt/β-catenin signaling pathway is essential for embryonic development and adult tissue homeostasis, consequently mutation of many of the components result in human birth defects, cancer and other diseases [1], [2]. In the absence of a Wnt ligand, the transcriptional co-activator β-catenin is continuously degraded in the cyptoplasm by a protein complex including the scaffolding protein Axin, tumor suppressor APC (adenomatous polyposis coli), GSK3 (glycogen synthase kinase 3) and CK1α (casein kinase 1α) [3]. The Axin complex mediates CK1α and GSK3 phosphorylation of β-catenin to provide a binding site for the β-Trcp E3 ubiquitin ligase, resulting in β-catenin ubiquitination and subsequent degradation. This process is inhibited when a Wnt ligand brings together two types of receptors: the Frizzled (Fz or FZD) serpentine receptors and the low-density lipoprotein receptor-related protein 5 or 6 (LRP5 or LRP6). The intracellular regions of FZD and LRP5 or LRP6 recruit the cytoplasmic proteins Dishevelled (DVL) and Axin, respectively [3]. Recruitment of Axin to the membrane by the Fz- LRP5/6 complex inhibits β-catenin phosphorylation and allows β-catenin levels to accumulate, resulting in β-catenin entering the nucleus and interacting with TCF/LEF (T cell factor/lymphoid enhancer factor) transcription factors to activate Wnt target gene transcription [4], [5].

LRP5 and LRP6 are the two LRP type of Wnt receptors in the human and mouse genome, and are both widely/ubiquitiously expressed [6], [7], [8], [9], [10]. Human LRP5 (1615 a.a.) and LRP6 (1613 a.a.) are 70% identical by paralogous conservation, and have a similar domain structure that consists of a large extracellular domain containing four β-propeller plus EGF repeats essential for binding to Wnt and other ligands/antagonists and three LDLR-A repeats [11]. The cytoplasmic region of LRP5/6 contains five highly conserved PPPSPxS motifs that serve as phosphorylation-regulated Axin binding sites [12], [13]. LRP5 has a central role in human bone mass regulation. Loss of function mutations in LRP5 result in osteoporosis-pseudoglioma (OPPG) primarily characterized by low bone mass [14]. On the other hand LRP5 “gain of function” missense mutations, which are clustered in the first β-propeller, cause high bone mass (HBM) disease [15], [16], likely as a result of disruption of binding and inhibition of LRP5 by its antagonists DKK1 and Sclerostin/SOST [17], [18]. Only one or two disease-causing LRP6 missense mutations have been found so far, including the one associated with coronary artery disease and osteoporosis [19], reflecting the likelihood that a severe or complete loss of function of LRP6 is incompatible with embryogenesis. Mouse models of the LRP5 [8], [20] and LRP6 [21] mutations recapitulate the human disorders. Lrp5−/− mice and heterozygous Lrp6+/− mice are viable and exhibit OPPG/osteoporosis phenotypes demonstrating their overlapping functions in at least some aspects of bone development/homeostasis [22]. However Lrp6−/− phenotypes are much more severe, as embryos resemble a composite phenotype of several canonical Wnt loss of function mutations and typically die during late stages of embryogenesis [7]. The severity of the Lrp6−/− phenotype is made worse by removing one or both alleles of Lrp5 and the resulting embryos die very early in development [23]. Indeed Lrp5/6 double knockout mutants fail to undergo gastrulation and closely resemble the phenotype of Wnt3 knockout mice [23], [24]. Collectively these data demonstrate that LRP6 has a more prominent role than LRP5 during development and that LRP5 and LRP6 together are essential for transducing Wnt/β-catenin signaling.

Other studies have peripherally compared the relative activities of LRP5 and LRP6. Expression of LRP6 alone, but not LRP5 alone, is able to induce a secondary axis in *Xenopus* embryos [25]. Similarly in mammalian cell culture, LRP5 only weakly activates the Wnt pathway in the absence of an exogenous Wnt, in contrast to the highly active LRP6 [25], [26]. On the other hand, coexpression of LRP5 and Wnt synergistically activates β-catenin signaling in both *Xenopus* embryos and mammalian cells [27]. These results are consistent with genetic studies that LRP6 appears to be the stronger of the two receptors in the Wnt pathway.

Given the importance of LRP5 and LRP6 in development and human diseases, we chose to dissect the molecular basis that distinguishes and regulates LRP5 and LRP6 functions by generating a series of LRP5/6 chimeric proteins. Our results suggest that despite highly conserved PPPSPxS motifs shared by both receptors, LRP6 cytoplasmic domain (LRP6C) harbors stronger signaling activity than that of LRP5 (LRP5C), likely owing to the fact that PPPSPxS motifs are more readily phosphorylated in LRP6 than in LRP5. We further identified between the two most carboxyl PPPSPxS motifs an intervening region that appears to account for most of the difference in phosphorylation and signaling capacity between LRP5C and LRP6C. Finally we show that LRP5/LRP6-binding to Axin is likely direct without GSK3 acting as an intermediary physical bridge.

## Results

### A predominant role of Lrp6 in Wnt signaling in MEFs

**Figure 1:**
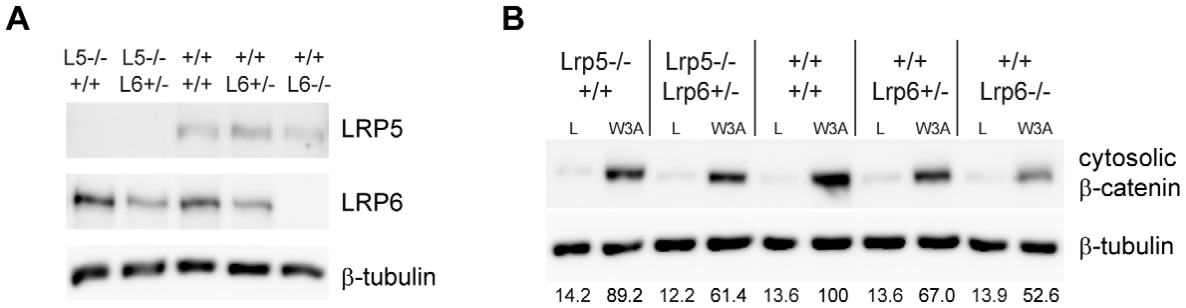
Lrp6−/− MEFs are less responsive than Lrp5−/− or WT MEFs to Wnt3a stimulation. (A) Endogenous expression of LRP5 and LRP6 in the mutant MEFS from total cell lysates. β-tubulin was used as a loading control. (B) Accumulation of cytoplasmic β-catenin in response to 6 hours of treatment of control L cell conditioned media (L) or L-Wnt3a conditioned media (W3A)examined via immunoblotting. Densitometry of cytosolic β-catenin levels, normalized to cytsolic β-tubulin loading control bands, are shown below each lane.

To compare the activity of the endogenous Lrp5 and Lrp6 we derived mouse embryonic fibroblasts (MEFs) from the wild-type (WT) +/+, Lrp5−/−, and Lrp6−/− embryonic day 14 (E14) embryos. We analyzed the expression of the endogenous receptors and confirmed the absence of protein in our knockout cells (Figure 1A). Furthermore we detected roughly half as much Lrp6 protein in MEFS derived from Lrp6+/− heterozygotes. We found that WT MEFs treated with Wnt3a conditioned media (CM) displayed a strong increase in cytosolic β-catenin levels compared to cells treated with the control CM (Figure 1B). Lrp5−/− MEFS, which only express the endogenous Lrp6 protein, displayed a slightly diminished response to Wnt3a compared to WT MEFs (Figure 1B). However Lrp6−/− MEFs, which only express the endogenous Lrp5 protein, exhibited a much weaker response to Wnt3a CM. We found similar results when using Wnt1 CM in the Lrp5−/− and Lrp6−/− knockout MEFS (data not shown). Additionally the Lrp5−/−; Lrp6+/− MEFS, which contained only one allele of Lrp6, displayed a higher Wnt response compared to Lrp6−/− cells containing two alleles of Lrp5 (Figure 1B). With the caveat that we do not know the abundance of Lrp5 or Lrp6 proteins on the cell surface, these results suggest that a majority of Wnt/β-catenin signaling in MEFs is mediated through Lrp6.

### LRP6C exhibits stronger signaling activity than LRP5C

**Figure 2:**
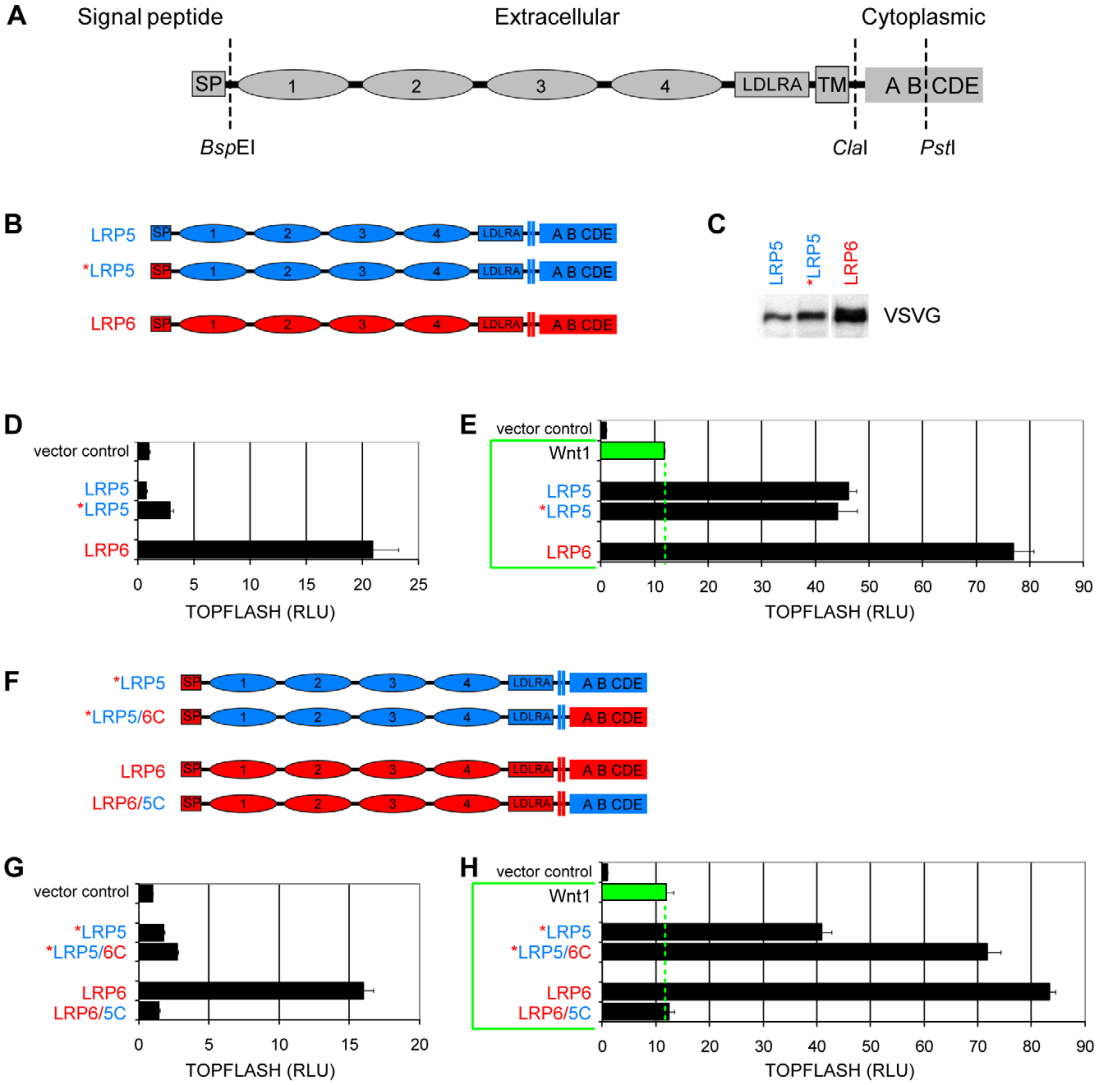
LRP5 and LRP6 cytoplasmic domains (LRP5C and LRP6C) show significant difference in signaling activities. (A) Schematic representation of LRP5/LRP6. SP: signal peptide; 1–4, propeller plus EGF-repeat 1 to 4; LDLRA: LDL receptor type A repeats; TM: transmembrane domain; A–E: PPPSPxS motifs A to E. *Bsp*EI was introduced together with the VSVG tag after the SP (Figure S1A); *Cla*I was introduced immediately after the TM domain and the endogenous *Pst*I is immediately after the motif B (Figure S1B). These restriction sites were used to generate chimeric receptors. (B) Schematic LRP5 (blue) and LRP6 (red) used for the signal peptide exchange experiment. (C) LRP5, *LRP5, and LRP6 levels in lysates from HEK293T cells that were transfected with the respective expression plasmid, as detected by a VSVG antibody. (D and E) TOPFLASH reporter activity of LRP5, *LRP5, and LRP6 alone (D) or co-expressed with Wnt1 (E) in HEK 293T cells. Wnt responsive TOPFLASH (firefly) luciferase units were internally controlled to the non-Wnt responsive Renilla reporter, and were normalized to vector control and presented as relative luciferase units (RLU). The dotted green line in E represents the activity of Wnt1 alone (through the endogenous receptor). (F) Schematic LRP5 (blue) and LRP6 (red) used for cytoplasmic domain swap experiments. (G and H) TOPFLASH reporter activity of chimeric LRP5/6 receptors expressed alone (G) or co-expressed with Wnt1 (H).

We set out to generate chimeric receptors between LRP5 and LRP6 to dissect their functional differences (Figure 2A and Figure S1A). In the process we found that the signal peptide of LRP5 is less active than that of LRP6, as *LRP5, in which the signal peptide of LRP6 replaced that of LRP5, exhibited a modest increase in the total protein level compared to the WT LRP5 (Figure 2B and 2C). Expression of *LRP5, but not of LRP5, showed a modest Wnt pathway activation in the Wnt responsive TOPFLASH luciferase assay, although this activation remained much weaker than that by expression of LRP6 alone (Figure 2D). In the presence of Wnt1 co-expression, however, *LRP5 and LRP5 exhibited virtually identical activity (Figure 2E). We speculate that under our experimental conditions of LRP5 or LRP5* over-expression, only a portion, but not all, of LRP5 or *LRP5 is activated by Wnt1, possibly due to availability of specific Fz proteins (and/or other factors such as LRP chaperones). For comparison of the mature LRP5 and LRP6 proteins (i.e., after the removal of the signal peptide) we made various LRP5/LRP6 chimeric proteins using *LRP5 and LRP6.

We first generated *LRP5/6C and LRP6/5C by swapping the cytoplasmic domain between *LRP5 and LRP6 (Figure 2F). Compared to *LRP5, *LRP5/6C displayed higher activity when expressed alone or in the presence of Wnt1 (Figures 2G and 2H). On the contrary LRP6/5C was much weaker and in fact was mostly inactive alone or in synergy with Wnt1 (Figure 2G and 2H). In repeating experiments LRP6/5C sometimes behaved as a dominant negative receptor such that the co-expression of LRP6/5C suppressed Wnt1-stimulated reporter activity. We noticed that the difference of signaling activity by over-expressed *LRP5 versus LRP6 in the absence of Wnt1 co-expression was drastic (about 8 folds, Figure 2G); in the presence of Wnt1 co-expression both *LRP5 and LRP6 were significantly activated but the difference between *LRP5 and LRP6 was quite smaller (about 2 folds, Figure 2H). One possibility is that under over-expression condition only a portion, but not all, of *LRP5 or LRP6 is activated by Wnt1 due to the availability of specific (and endogenous) Fz proteins. We also note the caveat that signaling by over-expressed *LRP5 or LRP6 alone may or may not be Fz-dependent.

**Figure 3:**
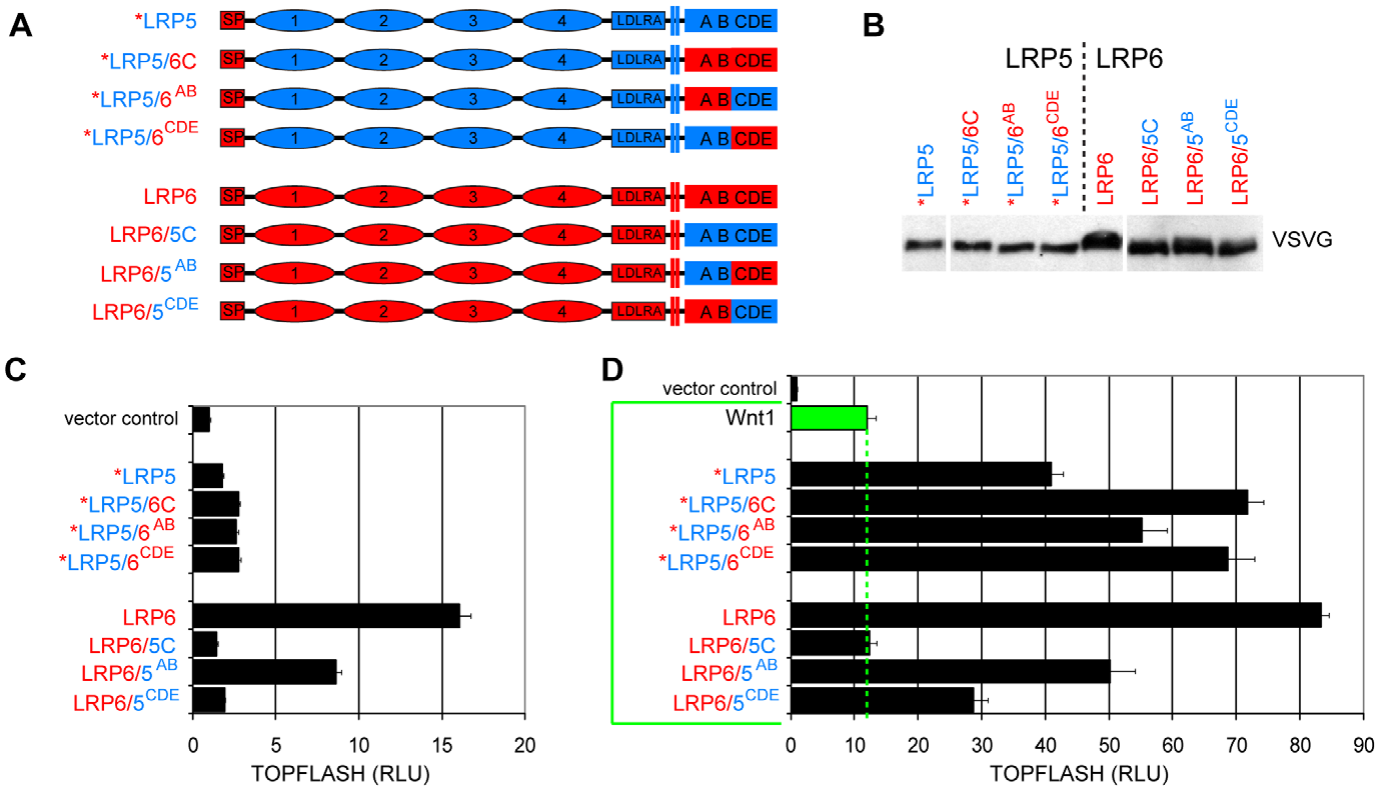
Additional analyses of the cytoplasmic domain of LRP5 and LRP6. (A) Schematic LRP5 (blue) and LRP6 (red) receptors used for the cytoplasmic domain swap experiment. (B) Chimeric LRP5/6 protein levels in lysates from HEK293T cells transfected with the respective expression plasmid, as detected by a VSVG antibody. (C and D) TOPFLASH reporter activity of chimeric LRP5/6 transfected alone (C) or co-transfected with Wnt1 (D).

To further map the activity difference between LRP5C and LRP6C, we utilized a conserved *Pst*I site located after PPPSPxS motif B in both LRP5 and LRP6 to divide the cytoplasmic domain into two parts, which contain motifs A plus B, and motifs C plus D plus E, respectively (Figure 3A and Figure S1B). These series of *LRP5 and LRP6 chimeric receptors were expressed at comparable levels, respectively (Figure 3B, data not shown). *LRP5/6^AB^ and *LRP5/6^CDE^, like *LRP5/6C, displayed higher activity compared to *LRP5 alone (Figure 3C). In the presence of Wnt1, *LRP5/6^CDE^ was similar to the *LRP5/6C, and both showed stronger activity than *LRP5/6^AB^, which in turn was more active than *LRP5 (Figure 3D). Reciprocally, we observed signaling activities in the following rank order: LRP6>LRP6/5^AB^ (which contained the C, D and E motifs of LRP6)>LRP6/5^CDE^ (which contained the A and B motifs of LRP6)>LRP6/5C (Figure 3C and 3D). We found similar results when LRP5 replaced *LRP5 in the generation of analogous chimeric receptors, and with either Wnt1 or Wnt3a (Figure S2A and S2B, and data not shown). These results together suggest that the first half (containing the A and B motifs) and the second half (containing the C, D, and E motifs) of LRP6 each confer more signaling activity than their LRP5 counterparts, and that the second half of LRP6C confers the strongest signaling activity. These results are consistent with our previous studies that LRP6 signaling is mostly mediated by C plus D plus E motifs [28], [29].

### Comparison of Axin interaction with phosphorylated LRP5 and LRP6

**Figure 4:**
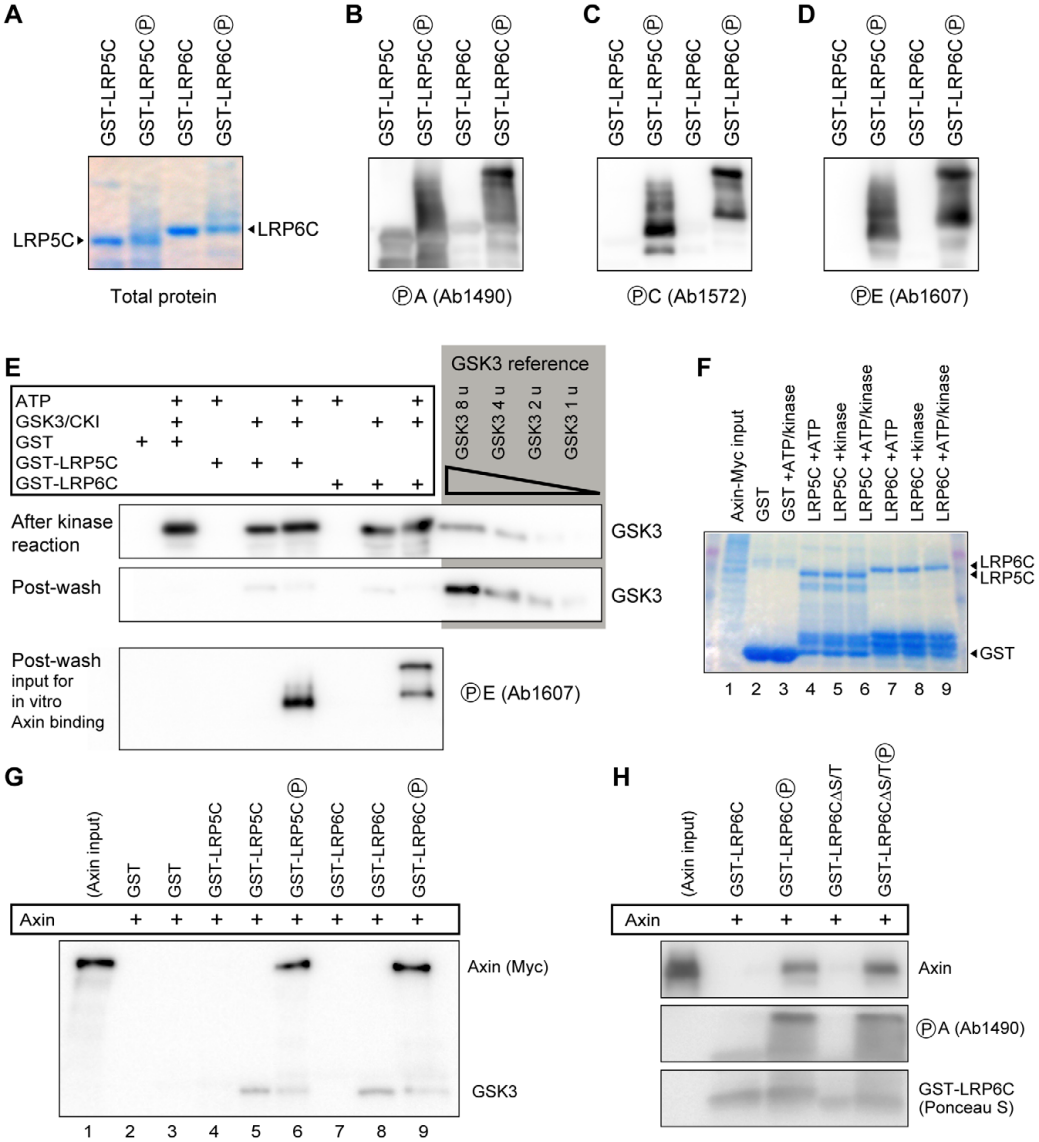
Phosphorylated LRP5 and LRP6 cytoplamic domain bind similarly and directly to Axin in vitro. (A–D) Purified GST-LRP5C and GST-LRP6C were phosphorylated in vitro at 30°C for 4 hours in the presence of recombinant GSK3 and CKI plus ATP. Total protein staining (SimplyBlue) (A) shows that phosphorylated LRP5C and LRP6C exhibit slower and smeary migration, as we previously reported for LRP6C [13]. Phosphorylation of LRP5C and LRP6C at motif A was detected via Ab1490 (B), at motif C via Ab1572 (C), and at motif E via Ab1607 (D). (E) Recombinant GSK3 can associate with LRP5C and LRP6C (but not GST) regardless of their phosphorylation status. GSK3 used to phosphorylate LRP5C and LRP6C was mostly washed away via high salt buffer prior to the in vitro Axin binding experiments. Top: GSK3 in the in vitro reaction prior to wash; middle: GSK3 post wash; bottom: LRP5C and LRP6C phosphorylation at motif E; right: two exposures of the same serial dilutions of GSK3 to show the relative amount of GSK3 bound to GST-LRP5C and GST-LRP6C. (F) Protein levels after the Axin binding experiment in (G), showing comparable amounts of GST, GST-LRP5C, and GST-LRP6C in different lanes. The protein bands around the GST size in lanes 4 to 9 likely represent partial proteolytic products. (G) Axin binds equivalently and specifically to phosphorylated LRP5C and LRP6C. Remaining recombinant GSK3 bound to LRP5C and LRP6C in the in vitro reaction is also shown. Immunoblotting was done with both anti-Myc (for Myc-tagged Axin) and anti-GSK3 antibodies. Note that Axin did not bind to unphosphorylated GST-LRP5C or –LRP6C even in the presence of LRP5/6C-bound GSK3. (H) Deletion of the entire serine/threonine-rich region in GST-LRP6CΔS/T (a 14-amino acid residue deletion, Figure S1B) had minimal effects on Axin binding to phosphorylated LRP6C.

Axin recruitment to activated/phosphorylated LRP5 or LRP6 has been well documented [30], [31], [32] and is believed to be a key step in Wnt signal transduction [3]. The significant difference between the Wnt signaling activity of LRP5C and LRP6C is surprising considering that the five PPPSPxS motifs, which are phosphorylation-dependent Axin docking sites, are virtually identical pair-wise between LRP5 and LRP6 (Figure S1B). We attempted to compare Axin binding to LRP6 and LRP6/5C, which exhibited highest and lowest signaling activities, respectively, via coimmunoprecipitation in cells. However as activated LRP6 signaling degrades the Axin protein as previously observed [33], we found that LRP6 expression resulted in much lower Axin levels compared to LRP6/5C expression (at both one and two days post-transfection, data not shown). Since these unequal Axin levels complicated the binding experiment, we chose to use a different in vitro approach that examines binding between Axin and recombinant LRP5C or LRP6C (fused with GST, glutathione S transferase). Previously we used this method to show that GST-LRP6C, upon phosphorylation by GSK3 plus CK1 in vitro, binds to Axin [13]. We purified GST-LRP5C (last 207 a.a. of LRP5) and GST-LRP6C (last 218 a.a. of LRP6), and subjected them to in vitro phosphorylation with recombinant GSK3 plus CKI in the presence (or absence) of ATP. Similar to phosphorylated GST-LRP6C [13], phosphorylated GST-LRP5C exhibited a slower migration pattern in gel electrophoresis (Figure 4A). Given the pair-wise conservation between PPPSPxS motifs of LRP5 and LRP6, we used phospho-specific antibodies that we have raised against motifs A, C, and E of LRP6 to examine the relative amount of phosphorylation of GST-LRP5C and GST-LRP6C. We detected similar intensities of phosphorylation at these three motifs with Ab1490 for phospho-motif A [12], Ab1572 for phospho-motif C [28], and Ab1607 for phospho-motif E [28] (Figure 4B–D). These antibodies by large were specific for phosphorylation of both LRP5 and LRP6 (Figure 4 B–D), although a minor exception was Ab1490, which cross-reacted slightly with unphosphorylated GST-LRP5C (Figure 4B). Nonetheless these experiments showed relatively comparable levels of phosphorylation of LRP5C and LRP6C by GSK3 and CK1 in vitro. Importantly, we found that phosphorylated, but not unphosphorylated, LRP5C and LRP6C each bound to the Axin protein from cell lysates at apparently comparable levels (lanes 6 and 9, Figure 4G). These data suggest that LRP5 and LRP6, if phosphorylated similarly, exhibit similar abilities to bind/recruit Axin.

### Axin-LRP5/6 association is unlikely bridged by GSK3

How phosphorylated/activated LRP5 or LRP6 binds to Axin remains somewhat debated. The initial finding that Axin binds to LRP5C in the yeast two-hydrid assay implied that the interaction might be direct [30], and phosphorylated PPPSPxS motifs in LRP6C were subsequently shown to mediate Axin-docking [12], [13], supporting a LRP5/6-Axin direct binding model. However since LRP6 can also bind to GSK3 [13], [34], possibly through PPPSP motifs that are substrates for GSK3 [13], [35], and/or through an upstream serine/threonine-rich region in LRP6 (Figure S1B) [36], [37], an alternative model was proposed that GSK3, which is known to complex with Axin, acts as a bridge between LRP6 and Axin [36]. We therefore designed experiments to test these two models for both LRP5 and LRP6. After phosphorylation of GST-LRP5C and GST-LRP6C by GSK3 plus CK1 we extensively washed these GST-fusion proteins on glutathione beads using a high salt condition (Figure 4E). Compared to input GSK3 and GSK3 reference standards, GST-LRP5C and GST-LRP6C proteins post washing retained a small amount of GSK3, which was independent of LRP5C/LRP6C phosphorylation and was not found in the lane of GST alone (Figure 4E), consistent with the direct and specific LRP6-GSK3 binding as previously reported [13], [34], [35]. However these LRP5/6-bound GSK3 proteins were not capable of recruitment of Axin into the complex (lanes 5 and 8, Figure 4G). The serine/threonine cluster upstream of PPPSPxS motifs was suggested to be a GSK3-binding site that can mediate LRP6C-GSK3-Axin interaction [36]. We generated a 14-amino acid deletion of this S/T cluster, LRP6CΔS/T (Figure S1B), which we found exhibited phosphorylation-dependent Axin binding that was indistinguishable from LRP6C (Figure 4H). Our results therefore do not support the model that GSK3 bridges LRP5/6-Axin association and are consistent with a direct LRP5/6-Axin binding through phopshorylated PPPSPxS motifs.

### Amino acid residues between PPPSPxS motifs D and E modulate LRP5/6 signaling activity

**Figure 5:**
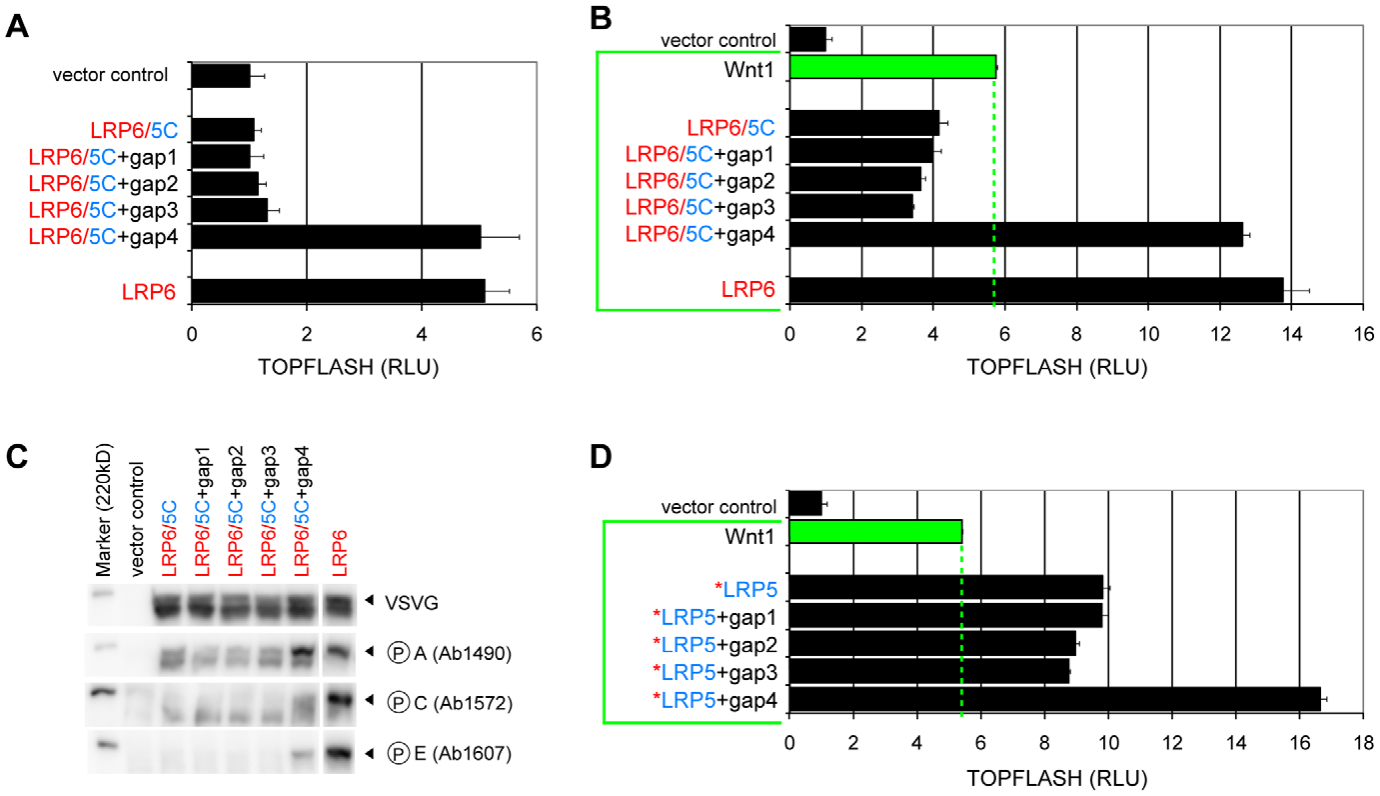
The gap4 region between the most carboxyl terminal PPPSPxS motifs regulates LRP5 activity and PPPSPxS phosphorylation. (A and B) TOPFLASH reporter activity of LRP6, LRP6/5C and its gap1–4 insertional derivatives transfected alone (A) or co-transfected with Wnt1 (B). (C) PPPSPxS phosphorylation at motifs A, C, and E of LRP6, LRP6/5C and its gap1–4 insertional derivatives, as detected via phosphorylation-specific antibodies. Receptor protein levels were detected via the VSVG antibody. The 220 kDa molecular weight marker is visible in the first lane. (D) TOPFLASH reporter activity of *LRP5 and its gap1–4 insertional derivatives that were co-transfected with Wnt1.

Our in vitro binding results suggested that when similarly phosphorylated, LRP5 is as effective as LRP6 in binding to Axin, consistent with the high conservation of the five PPPSPxS motifs found in LRP5 and LRP6. However in the context of the full-length receptor, LPR5C is much less effective in activating Wnt signaling. One possible explanation is that LRP6 is more readily phosphorylated than LRP5 in vivo, and the amino acid residues outside the conserved PPPSPxS motifs may be responsible for the observed functional difference. An alignment of LRP5C and LRP6C from multiple vertebrate species reveals several gaps, referred to here as gaps1–4, where LRP6 contains additional amino acid residues between PPPSPxS motifs (Figure S1B). We first introduced these gap amino acid residues into LRP6/5C, which behaved inactive or slightly dominant negative. Addition of Gap1, Gap 2 or Gap3 did not significantly alter the activity of LRP6/5C (Figure 5A). To our surprise, the addition of the gap4 serine residue between motifs D and E in the LRP6/5C+gap4 mutant resulted in high signaling activity that was comparable to LRP6 (Figure 5A). LRP6/5C+gap4 also resembled LRP6 when co-expressed with Wnt1, while LRP6/5C or each of LRP6/5C+gap1/2/3 derivatives behaved inactive (in fact dominant negative) (Figure 5B). Importantly, we detected phosphorylation at motifs A, C, and E in LRP6 and LRP6/5C+gap4, but not in LRP6/5C or LRP6/5C+gap1/2/3 (Figure 5C). These data are consistent with the idea that LRP5C is less active in vivo due to poorer phosphorylation, and demonstrate that introduction of gap4 is sufficient, at least in part, to enhance LRP5 phosphorylation and signaling. We next introduced these gaps in the full length *LRP5. *LRP5+gap4, but none of the *LRP5+gap1/2/3 derivatives, resulted in elevated signaling activity with Wnt1 to a level comparable to that of LRP6 (Figure 5D).

**Figure 6:**
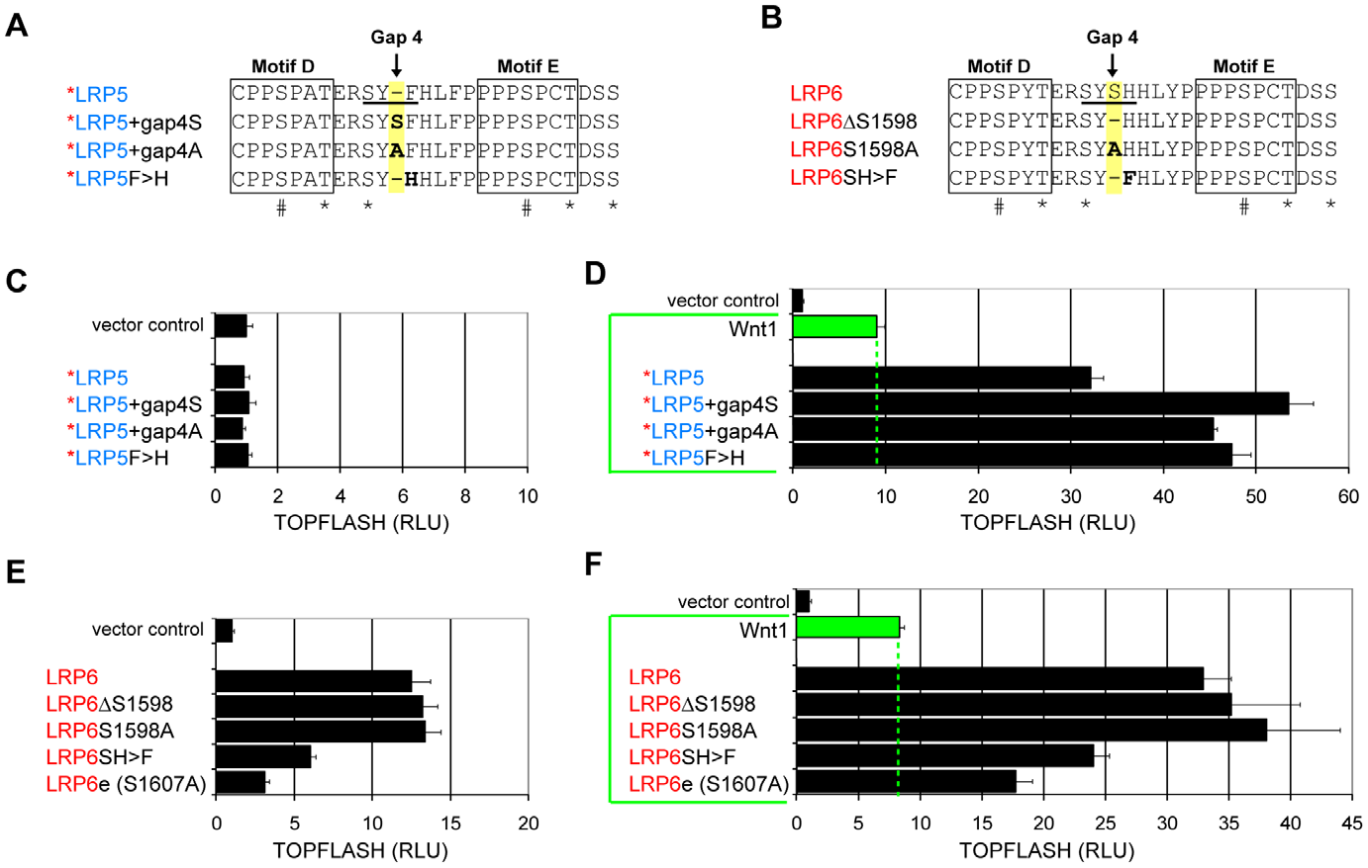
The context and spacing of the gap4 region together reciprocally regulate LRP5 and LRP6 signaling activities. (A and B) Additional derivatives in the gap4 region (underlined) of *LRP5 and LRP6. The mutated residue of each derivative is in bold. Motifs D and E are boxed and known sites phosphorylated by GSK3 (#) and CKI (*) are indicated below the alignment. The LRP6 gap4 serine is not predicted to be a CKI phosphorylation site. (C–D) TOPFLASH reporter activity of *LRP5 and derivatives alone (C) or co-transfected with Wnt1 (D). (E–F) TOPFLASH reporter activity of LRP6 and derivatives alone (E) or co-transfected with Wnt1 (F). The LRP6e mutant [28], which has PPPAP instead of PPPSP in motif E, was used for comparison.

The serine residue in the gap4 region was intriguing and hinted the possibility of regulation via post-translational modifications such as phosphorylation. However this seemed unlikely as an alanine insertion in the place of the serine residue still conferred significant signaling enhancement in the *LRP5+gap4A mutant (Figure 6D). Further inspection of the gap4 region in LRP5, SYF, versus SYSH in LRP6 (Figure 6A and 6B), prompted us to generate *LRP5F>H, in which the phenylalanine (LRP5 residue 1601) was replaced by histidine. *LRP5F>H also exhibited significant higher activity than *LRP5 (Figure 6D). We thus considered the scenario that the tyrosine residue and its possible phosphorylation in gap4 may be differentially regulated in LRP5 versus LRP6 due to the neighboring F versus H change (Figure 6A, 6B and Figure S1B). But this also seemed unlikely because replacement of the tyrosine by phenylalanine had little effect on signaling by either *LRP5 or LRP6 (data not shown). These results suggest that the collective context of gap4 residues plays a major role in the activity difference between LRP5 and LRP6.

We generated reciprocal changes in LRP6. Removing gap1 or 2 or 3 in LRP6 did not affect its signaling function (data not shown), mirroring the little effect of insertion of these gaps each in LRP5 (Figure 5A and 5B). Similar to the *LRP5 data that the serine residue per se in gap4 is not essential, a deletion of serine 1598 or its replacement by alanine in LRP6ΔS1598 and LRP6S1598A, respectively, did not affect LRP6 signaling (Figure 6E and 6F). Importantly however, a substitution of serine 1598 and histidine 1599 in LRP6 gap4 with phenylalanine, generating the LRP6SH>F mutant that had a gap4 resembling that in LRP5, significantly diminished LRP6 signaling activity (Figure 6E and 6F). Note that the reduction of activity in LRP6SH>F was on par to that observed in the LRP6e mutant, which has a non-functional PPPAP alteration in motif E, and was much more pronounced than that observed in LRP6a or LRP6b mutants harboring a non-functional PPPAP alteration in motif A and B, respectively (Figure 6E and 6F) [28]. Thus the gap4 contribution to LRP6 signaling is similar to that by PPPSPxS motif E but more significant than that by motif A or B. These reciprocal data in LRP5 and LRP6 demonstrate that the gap4 region between the last two PPPSPxS motifs (D and E) accounts for most of the functional difference between the cytoplasmic domain of these two Wnt receptors.

## Discussion

The LRP5 and LRP6 genes likely arose from duplication of a single ancestral gene approximately 500 million years ago during the emergence of the chordate phylum. Since then LRP5 and LRP6 have retained a relatively high degree structural similarity and paralogous conservation, resulting in partially redundant functions as Wnt receptors in vertebrates. However since this early gene duplication, it is likely that LRP5 and LRP6 have independently evolved to the receptors they are today in the human genome. Genetic studies suggest that LRP6 has a more dominant role in Wnt signal transduction [3]. Consistent with the severity of the developmental phenotypes of Lrp5 and Lrp6 mutant mice [22], [23], our comparison of the MEFs deficient for Lrp5 or Lrp6 indicates that a majority of Wnt/β-catenin signaling is mediated through Lrp6 (Figure 1). Moreover we were able to show a dose dependent correlation of Wnt responsiveness in MEFS containing two, one or no functioning Lrp6 alleles. Our data in MEFs in general are consistent with the previous genetic analyses of Lrp5 and Lrp6 compound mutant mice with different allelic combinations [22], [23]. Further evidence of the critical importance of LRP6 is found through interspecies protein comparisons. The orthologous conservation of human LRP6 to mouse (98%), chicken (92%) and *Xenopus* (85%) Lrp6 is greater than human LRP5 to mouse (94%), chicken (88%) and *Xenopus* (80%) Lrp5 (Percentage numbers represent amino acid sequence identity based on BLAST2 comparisons, data not shown), indicating that LRP6 is more conserved during evolution whereas changes in LRP5 may be more tolerable to the organism. These observations may also in part explain the small number of identified human LRP6 mutations in the literature compared to those of LRP5 [19], [38]. In this study we aimed to understand the molecular underpinning that accounts for different LRP5 and LRP6 signaling activities for two considerations. First, both LRP5 and LRP6 are key therapeutic targets for treatment of human diseases including cancer and/or osteoporosis; and secondly, LRP5 and LRP6 share five virtually identical orthologous PPPSPxS motifs, implying that other unknown mechanisms may operate to tune the Wnt receptor activities through these motifs.

By generating a series of reciprocal chimeric receptors between LRP5 and LRP6, we found that difference between the cytoplasmic domain of LRP5 and LRP6 underlies, to a significant degree, the different signaling activities of these two receptors (Figure 2). Our previous comprehensive analysis has revealed that the five PPPSPxS motifs, from A to E (Figure S1B), contribute to LRP6 signaling in the rank order of C>D = E>A>B, with the carboxyl C, D, and E cluster making up most of the LRP6 signaling output [28]. Comparing LRP5 and LRP6, we found that motifs A plus B in LRP6 have stronger activity than the corresponding A and B pair in LRP5, while similarly the cluster of motifs C and D and E of LRP6 is more potent than that of LRP5 (Figure 3 and Figure S2). Most strikingly, our analysis identified between motifs D and E a short intervening region, termed gap4, which has significant and reciprocal effects on LRP5 and LRP6 activities. Thus LRP5 with an altered and “LRP6-like” gap4 exhibits stronger signaling comparable to LRP6, whereas LRP6 with an altered and “LRP5-like” gap4 region exhibits weaker signaling similar to LRP5 (Figures 5 and 6). Indeed our data suggest that the contribution by gap4 to LRP6 signaling is on par to that by motif E and is more prominent than that by motif A or B (Figure 6) [28], [29].

It has been established that Axin binds to both LRP5 [30] and LRP6 [32], and that phosphorylated PPPSPxS motifs provide Axin-docking sites that mediate LRP6 signaling [12]. The difference between LRP5 and LRP6 signaling could therefore be a result of their different Axin-binding properties. But we found that recombinant LRP5C and LRP6C, upon in vitro phosphorylation by GSK3 and CK1, exhibit indistinguishable phosphorylation-dependent binding to Axin (Figure 4), suggesting that the difference of LRP5 and LRP6 signaling in vivo is likely at a step prior to Axin-binding, i.e., at how effectively the PPPSPxS motifs are phosphorylated. Fully consistent with this possibility, we found that, using antibodies specific for phospho-A, -C, and –E, LRP6 is more readily phosphorylated at these PPPSPxS motifs than its LRP5 counterparts (Figure 5). More revealingly, the gap4 region in LRP5 when altered to resemble that in LRP6 not only enhances LRP5 signaling but also LRP5 phosphorylation at these PPPSPxS motifs (Figure 5C). On the other hand other gap regions (gaps1, 2, and 3) have minimal effects on LRP5 signaling or phosphorylation (Figure 5). Thus we suggest that the difference in LRP5 and LRP6 signaling activity is largely due to their effectiveness of phosphorylation at PPPSPxS motifs, and we have identified the gap4 region that is responsible, at least in a significant part, for the difference between LRP5 and LRP6 phosphorylation and signaling.

The gap4 region lies between motifs D and E, but it has a strong effect on phosphorylation of not only motif E (and possibly D), but also of motifs C and A that are some distance away (Figure 5C and Figure S1B). We believe that this is consistent with the “LRP6 signal amplification” model we previously proposed [28], which corroborates genetic observations in *Drosophila* embryos [39]. This model is based on the observation of a local positive feed forward loop between Axin and LRP6 PPPSPxS motifs. Thus Axin not only binds to phosphorylated PPPSPxS motifs, but also promotes and is required for PPPSPxS phosphorylation via its recruitment of the Axin-GSK3 complex [28], [40]. Therefore phosphorylation of one PPPSPxS motif has a stimulatory effect on that of other PPPSPxS motifs. Indeed we previously demonstrated that LRP6 phosphorylation at C or E profoundly relies on the presence of other PPPSPxS motifs [28]. Here we interpret that the gap4 region, by directly regulating phosphorylation at motif E (and possibly motif D) nearby, exerts significant effects on phosphorylation of other motifs such as A and C (Figure 5) through such a signal amplification mechanism. This may also explain why gap4 has a prominent role in the overall LRP5 and LRP6 signaling output.

The gap4 sequence SYF is conserved among vertebrate Lrp5 proteins, and is distinct from SYSH in gap4 that is invariable among vertebrate Lrp6 orthologs (Figure S1B). Such a conserved sequence difference together with its critical modulation of LRP5 and LRP6 signaling activity are unlikely to be co-incidental. Although the serine and tyrosine residues in gap4 call for potential (and differential) phosphorylation regulation in LRP5 and LRP6, our mutational analyses do not seem to support such a scenario (Figure 6 and data not shown). How does gap4 regulate phosphorylation at PPPSPxS motifs and thereby LRP5/LRP6 signaling remains to be investigated. One possibility is that gap4 in LRP5 or LRP6, perhaps in conjunction with flanking residues, serves as a binding site for an unknown protein. Alternatively the sequence difference between LRP5 and LRP6 results in conformational difference that affects relative positioning of the last two PPPSPxS motifs (D and E), impacting signal amplification. Intriguingly the predicted secondary structure of LRP6 gap4 region contains a “turn” that is lacking in LRP5, and furthermore. the LRP5+gap4S mutant gains, while the LRP6 SH>F mutant loses, this predicted turn (Figure S3), correlating with the increase and decrease of receptor signaling strength, respectively.

We note that while other gap regions, including gap2, do not appear to influence LRP5/6 activities in Wnt/β-catenin signaling, a recent study has shown that the RMTSV region of LRP6 gap2 serves as a potential phosphorylation site (T1558) for Protein Kinase A (PKA) and mediates LRP6 and Gα_s_ interaction in response to parathyroid hormone (PTH) binding to LRP6 [41]. This PTH responsive PKA site in gap2 is absent in Lrp5 (Figure S1B). Although our data do not favor a similar phosphorylation-dependent regulation of gap4, this and our studies together highlight important regulatory roles of different gap regions between PPPSPxS motifs in LRP5 and LRP6 in Wnt and possibly other signaling pathways.

Our study further helps to resolve a controversial issue regarding LRP5/6-Axin interaction. Earlier findings based on yeast two-hybrid assays suggested that LRP5/6-Axin association is likely direct [30] and is mediated through phosphorylated PPPSPxS motifs as Axin-docking sites [12], [13], although this model has not ruled out the caveat that GSK3-like proteins in yeast may have a role in mediating the two-hydrid interaction. This direct interaction model is complicated/challenged by the findings that GSK3 also binds to LRP6 and performs PPPSP phosphorylation [13], [34], [36], [37], and that phosphorylated PPPSPxS peptides can inhibit GSK3 phosphorylation of β-catenin presumably through direct interaction with GSK3 [35], [36]. Furthermore the serine/threonine-rich region upstream of the PPPSPxS motif A (Figure S1B) may also bind to GSK3 [36], [37]. Given these scenarios and the established Axin-GSK3 interaction, a recent in vitro study has argued that Axin-LRP6 interaction is indirect and requires GSK3 as an intermediate bridge [36]. Using recombinant GST-LRP5C or -LRP6C plus GSK3 (and CK1) we performed in vitro reconstitution of phosphorylation-dependent LRP5C/LRP6C-Axin interaction. We found that GSK3 indeed binds to GST-LRP5C and -LRP6C, but not to control GST, this binding is however independent of LRP5C/LRP6C phosphorylation (Figure 4). By contrast, Axin binds only to GST-LRP5C and -LRP6C that have been phosphorylated by GSK3 plus CK1 (Figure 4). Importantly the presence of GSK3 with LRP5C/LRP6C does not result in Axin recruitment (Figure 4G). Furthermore, LRP6CΔS/T, which harbors a deletion of the serine/threonine-rich region that was suggested to be a GSK3-binding site [36], [37], binds to Axin in a phosphorylation-dependent manner that is indistinguishable to the wild type LRP6C (Figure 4H). Therefore our results are consistent with a direct LRP5/6-Axin interaction and do not support the model that GSK3 is the intermediate bridge between LRP5/6 and Axin. We note that in experiments supporting the GSK3-bridging model [36], the authors employed an Axin fragment that lacks the so-called DIX domain, which was suggested to be required for LRP5/6-Axin interaction [30].

In summary we have determined that the cytoplasmic domain of LRP5 and LRP6 plays a major role in the different signaling activity of these two Wnt receptors, and identified between the last two carboxyl PPPSPxS motifs an intervening gap4 region that has a key modulatory function in LRP5/LRP6 phosphorylation and signaling output. We have also provided evidence that argues for direct LRP5/LRP6-Axin interaction. Collectively our data provide significant new insights into the molecular mechanism of LRP5/LRP6 in Wnt signal transduction in development and human diseases.

## Materials and Methods

### Plasmids

Human LRP5 and LRP6 were tagged with the VSVG epitope and cloned into pCS2+ as previously described [12]. When not present in the endogenous sequence, unique restriction enzyme sites were introduced using the QuickChange Mutagenesis Kit (Stratagene). Point mutants and c-terminal insertion/deletion mutations were created in the LRP5ΔN and LRP6ΔN constructs, and later transferred over to the full-length receptor using the *Cla*I site before the transmembrane domain. LNCX-Wnt1, LNCX-Wnt3a or an empty LNCX vector, was used for co-expression assays. GST-LRP5C (amino acids 1409–1615) was cloned into pGEX4 using similar boundaries based on GST-LRP6C (amino acids 1396–1613). GST-LRP6CΔS/T contains a 14 amino acid deletion (Δ1466–1479, see Figure S1B). For experiments analyzing full-length LRP5/6 phosphorylation, pCS2-MESD was added at a 10∶1 LRP5/6∶MESD ratio to facilitate processing and trafficking of the receptors to the membrane. Full details of all plasmids are available upon request.

### Dual luciferase assay

Mammalian cell transfections were done in HEK 293T cells (ATCC# CRL-11268) using FuGENE 6 and performed in triplicate. Cells were plated at 1×10^5^/ml in 24 well plates and transfected the following day with a total of 300 ng of DNA/well [50 ng TOPFLASH, 10 ng TK-*Renilla*, 90 ng pCS2+ (empty vector), 50 ng of LNCX/Wnt1 and 100 ng of LRP5/6 constructs or pCS2 vector control]. Dual luciferase reporter assays were performed as previously described [28]. Representative results are shown from one of three (or more) independent experiments.

### In vitro phosphorylation and binding assays

Recombinant GSK3 (GSK3β: P6040, NEB) and CKI (CKIδ: P6030, NEB) were used for in vitro phosphorylation and binding assays as previously described [13]. Following the in vitro phosphorylation reaction with 250 units of GSK3 and 500 units of CKI, GST proteins were thoroughly washed to minimize the amount of recombinant GSK3 and CKI in the future binding assay (4× using a high salt condition: 50 mM HEPES pH 7.4, 500 mM NaCl, 1.5 mM EDTA, 1% Triton X-100, 10% glycerol). The same HEPES buffer containing only 150 mM NaCl was used for Axin binding conditions and post-binding washes (6×).

### Immunoblotting and antibodies

Polyclonal anti-VSVG (V4888, Sigma) was used to detect the expressed LRP5/6 chimeric receptors by western blot analysis as previously described [28]. MEFS were generated from E13.5 mouse embryos (*Lrp5* null [23] and *Lrp6* null [7]). Individual cell lines were established and immortalized using large T antigen. No detergents were present in the lysis buffer for analysis of cytosolic β-catenin levels (610153, BD) with β-tubulin (E7, DSHB) as a loading control. MutliGauge analytic software (Fujifilm) was used to calculate cytoplasmic β-catenin levels normalized to β-tubulin. Total cell lysates were used to detect endogenous LRP5 (D80F2, Cell Signaling) and LRP6 (C47E12, Cell Signaling) in MEFS. GSK3 was detected using a monoclonal antibody (4G-1E, Millipore) and Axin-Myc was detected using either a monoclonal anti-Myc antibody (9E10, Santa Cruz) or polyclonal anti-Axin antibody (34–5900, Invitrogen) [13]. Phospho-specific LRP6 antibodies to the A site (Ab1490, Cell Signaling), C and E sites (Ab1572 and Ab1607, previously described [28]) were used to verify in vitro phosphorylation of GST-LRP5C and GST-LRP6C. Total protein was detected using SimplyBlue (Invitrogen) or Ponceau S (Sigma).

## Supporting Information

Figure S1 **Location of VSVG epitope tag and alignment of the LRP5 and LRP6 cytoplasmic domains.** (A) The predicted signal peptide cleavage site for LRP5 and LRP6. Insertion of a VSVG epitope tag and *Bsp*EI site is a few amino acid residues after the cleavage site. Underlined residues represent the beginning of the first YWTD β-propeller structure. (B) ClustalW alignment of LRP5 and LRP6 proteins from human, chicken, frog, and zebrafish. Amino acid residues highlighted in black, grey, and light grey represent identical, conservative, and similar amino acids, respectively. Green boxes indicate PPPSPxS motifs A, B, C, D and E. The conserved *Pst*I site located after motif B in LRP5 and LRP6 cDNAs was used to create the LRP^AB^/^CDE^ swap constructs. The extra amino acid residues in LRP6 between the PPPSPxS motifs compared to LRP5 in gaps 1–4 are highlighted in yellow. Deleted residues in GST-LRP6CΔS/T, Δ1466–1479, are shown with asterisks. Proteins used for alignment: LRP5, Human (NP_002326, 1407–1615), *Gallus gallus* (NP_001012915, 1408–1616), *Xenopus laevis* (NP_001079163, 1397–1605), and *Danio rerio* (NP_001170929, 1223–1430); LRP6: Human (NP_002327, 1394–1613), *Gallus gallus* (XP_417286, 1349–1567), *Xenopus laevis* (NP_001079233, 1394–1613), and *Danio rerio* (NP_001128156, 1398–1620).
(TIF)

Figure S2 **Comparison of the LRP5 and LRP6 cytoplasmic domains using the endogenous LRP5 signal peptide.** TOPFLASH reporter activity of LRP5/6 and LRP6/5 chimeric receptors co-transfected with Wnt1 (A) or Wnt3a (B). The dotted line represents the activity of Wnt alone (through the endogenous receptor).
(TIF)

Figure S3 **Secondary structure prediction for LRP5 and LRP6 cytoplamic domain containing the most carboxyl terminal PPPSPxS motifs D and E (boxed) and the gap4 region in between (underlined).** Garnier-Robson secondary structure prediction [42] was performed via Protean from DNASTAR Lasergene8 for full-length Human LRP5 and LRP6 using default parameters. The gap4 region of LRP6, but not LRP5, has a predicted turn. The LRP5+gap4S mutant (more active than LRP5) harbors a predicted turn, whereas the LRP6 SH>F mutant destroys the turn and thus resembles LRP5. Neither LRP5 nor LRP6 has any α helix predicted for this span (not shown).
(TIF)
