# Supplementary figures and images for: Dissecting Molecular Differences between Wnt Coreceptors LRP5 and LRP6
Source: PLoS One. 2011 Aug 24;6(8):e23537. doi: 10.1371/journal.pone.0023537 (PMC3160902; doi:10.1371/journal.pone.0023537)

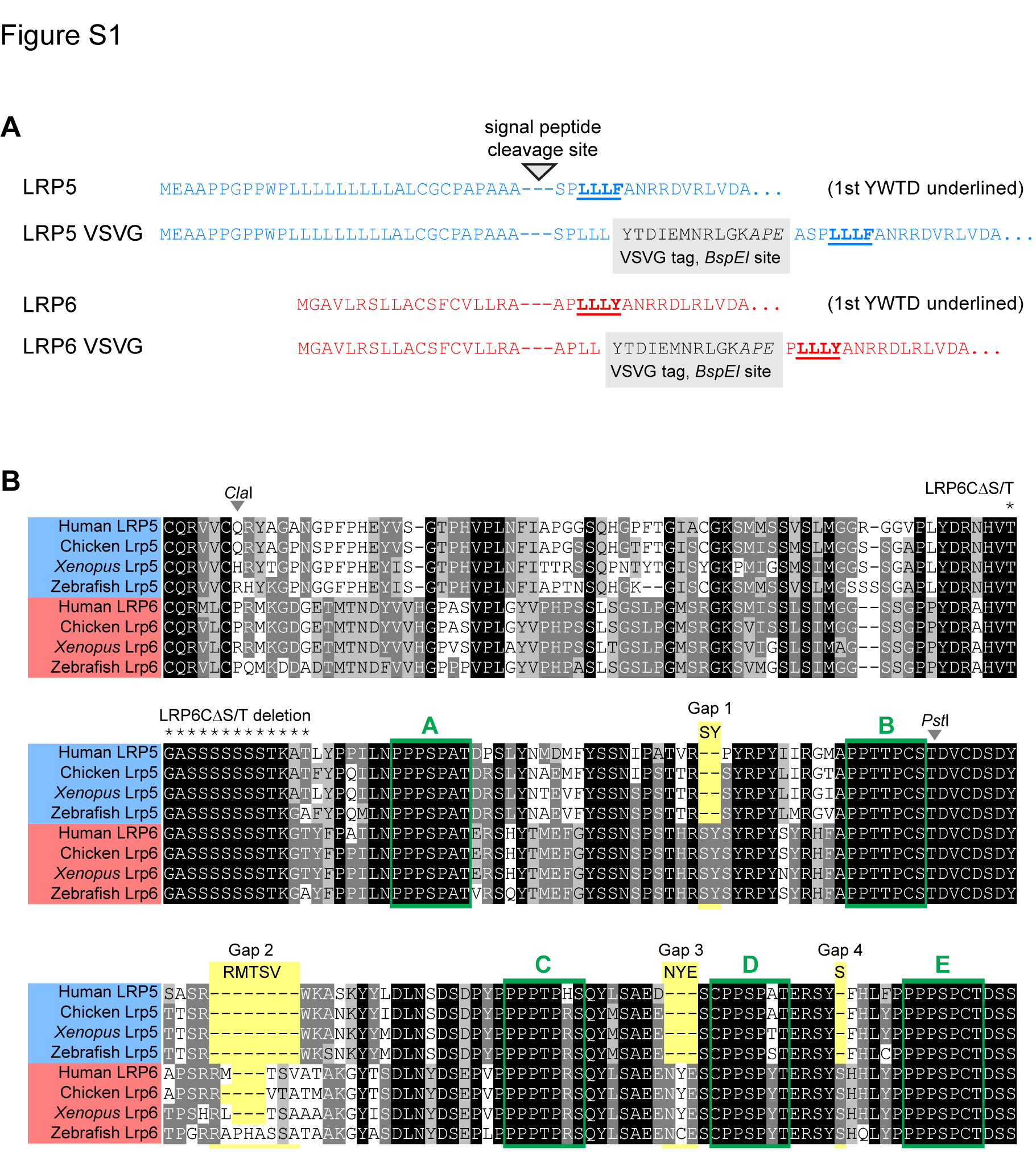

Supplement: Figure S1 — Location of VSVG epitope tag and alignment of the LRP5 and LRP6 cytoplasmic domains. (A) The predicted signal peptide cleavage site for LRP5 and LRP6. Insertion of a VSVG epitope tag and BspEI site is a few amino acid residues after the cleavage site. Underlined residues represent the beginning of the first YWTD β-propeller structure. (B) ClustalW alignment of LRP5 and LRP6 proteins from human, chicken, frog, and zebrafish. Amino acid residues highlighted in black, grey, and light grey represent identical, conservative, and similar amino acids, respectively. Green boxes indicate PPPSPxS motifs A, B, C, D and E. The conserved PstI site located after motif B in LRP5 and LRP6 cDNAs was used to create the LRPAB/CDE swap constructs. The extra amino acid residues in LRP6 between the PPPSPxS motifs compared to LRP5 in gaps 1–4 are highlighted in yellow. Deleted residues in GST-LRP6CΔS/T, Δ1466–1479, are shown with asterisks. Proteins used for alignment: LRP5, Human (NP_002326, 1407–1615), Gallus gallus (NP_001012915, 1408–1616), Xenopus laevis (NP_001079163, 1397–1605), and Danio rerio (NP_001170929, 1223–1430); LRP6: Human (NP_002327, 1394–1613), Gallus gallus (XP_417286, 1349–1567), Xenopus laevis (NP_001079233, 1394–1613), and Danio rerio (NP_001128156, 1398–1620). (TIF) [file pone.0023537.s001.tif]

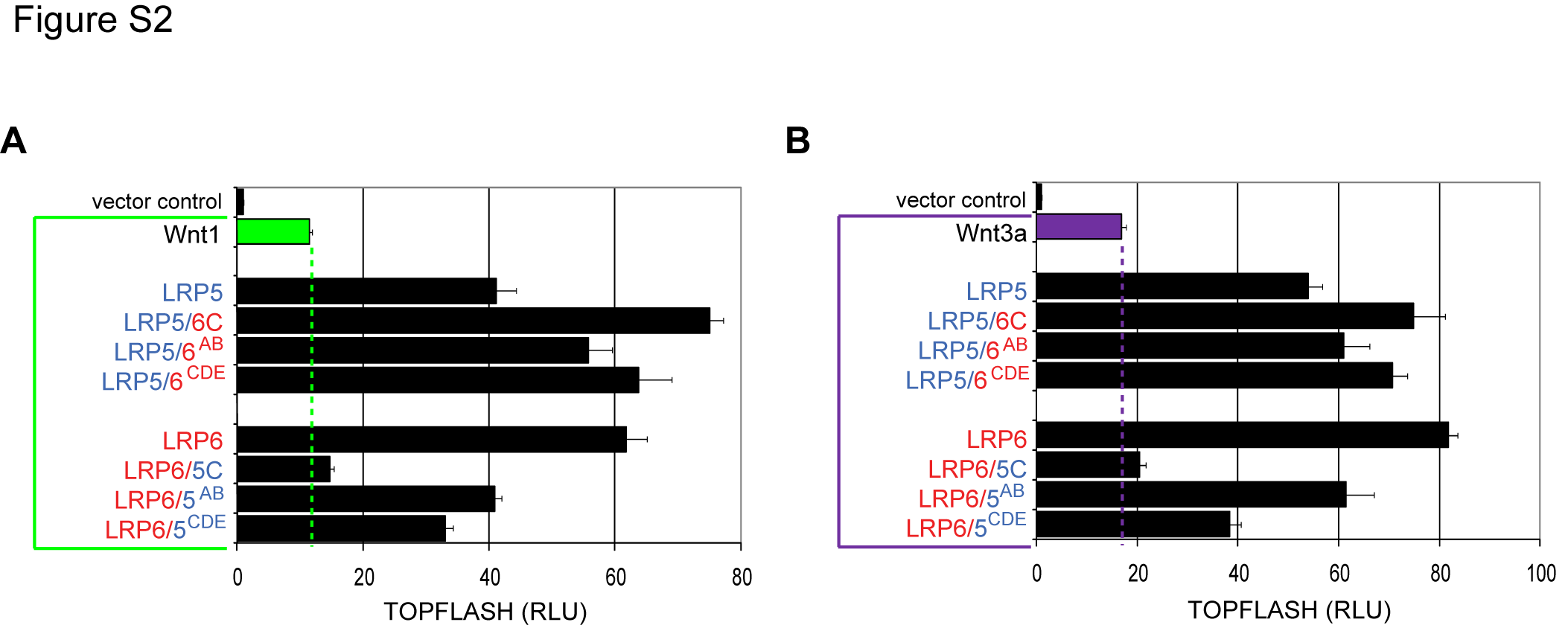

Supplement: Figure S2 — Comparison of the LRP5 and LRP6 cytoplasmic domains using the endogenous LRP5 signal peptide. TOPFLASH reporter activity of LRP5/6 and LRP6/5 chimeric receptors co-transfected with Wnt1 (A) or Wnt3a (B). The dotted line represents the activity of Wnt alone (through the endogenous receptor). (TIF) [file pone.0023537.s002.tif]

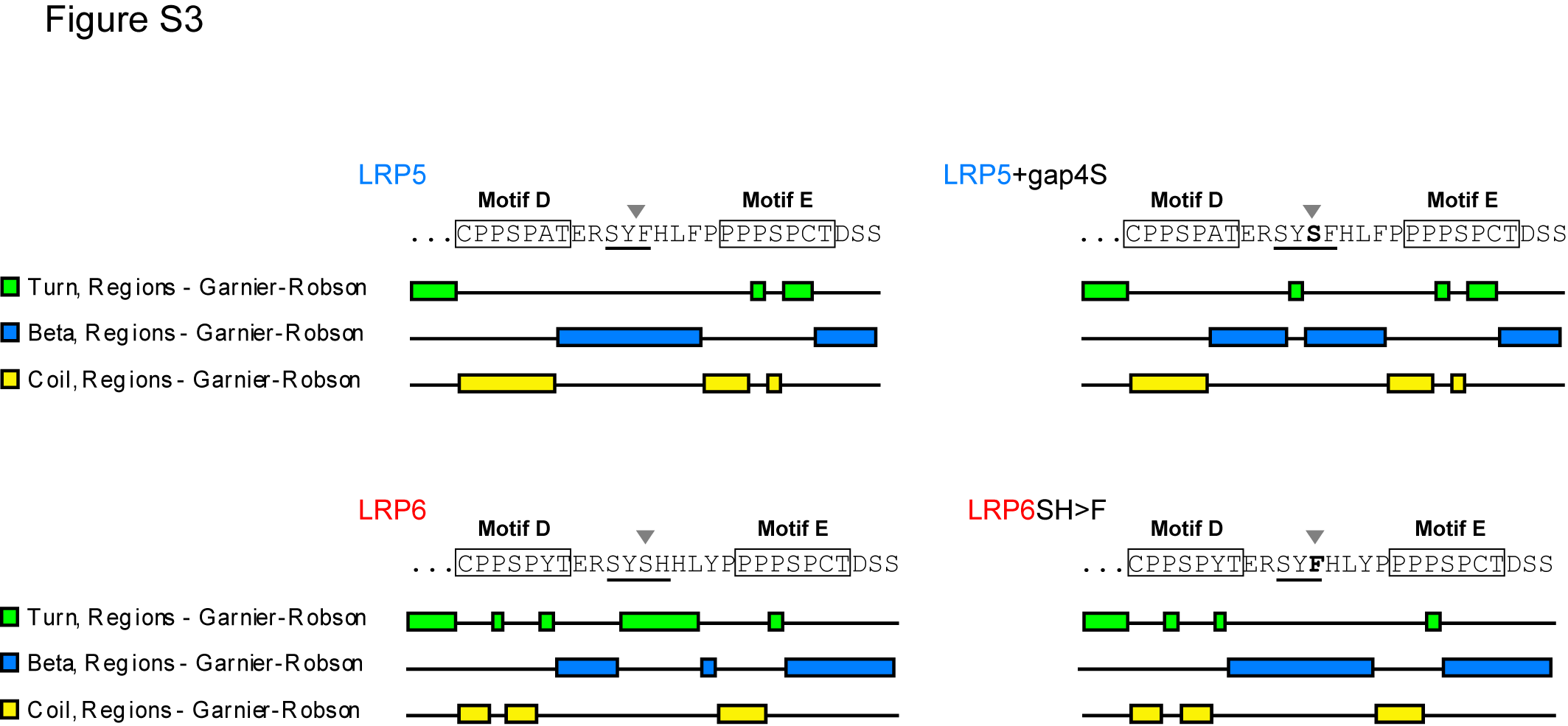

Supplement: Figure S3 — Secondary structure prediction for LRP5 and LRP6 cytoplamic domain containing the most carboxyl terminal PPPSPxS motifs D and E (boxed) and the gap4 region in between (underlined). Garnier-Robson secondary structure prediction [42] was performed via Protean from DNASTAR Lasergene8 for full-length Human LRP5 and LRP6 using default parameters. The gap4 region of LRP6, but not LRP5, has a predicted turn. The LRP5+gap4S mutant (more active than LRP5) harbors a predicted turn, whereas the LRP6 SH>F mutant destroys the turn and thus resembles LRP5. Neither LRP5 nor LRP6 has any α helix predicted for this span (not shown). (TIF) [file pone.0023537.s003.tif]
